# How contrast situations affect the assignment of causality in symmetric physical settings

**DOI:** 10.3389/fpsyg.2014.01497

**Published:** 2015-01-08

**Authors:** Sieghard Beller, Andrea Bender

**Affiliations:** Department of Psychosocial Science, Faculty of Psychology, University of BergenBergen, Norway

**Keywords:** causal cognition, physical domain, causal judgments, framing effect, contrast effect, content effect

## Abstract

In determining the prime cause of a physical event, people often weight one of two entities in a symmetric physical relation as more important for bringing about the causal effect than the other. In a broad survey (Bender and Beller, 2011), we documented such weighting effects for different kinds of physical events and found that their direction and strength depended on a variety of factors. Here, we focus on one of those: adding a *contrast situation* that—while being formally irrelevant—foregrounds one of the factors and thus frames the task in a specific way. In two experiments, we generalize and validate our previous findings by using different stimulus material (in Experiment 1), by applying a different response format to elicit causal assignments, an analog rating scale instead of a forced-choice decision (in Experiment 2), and by eliciting explanations for the physical events in question (in both Experiments). The results generally confirm the contrast effects for both response formats; however, the effects were more pronounced with the force-choice format than with the rating format. People tended to refer to the given contrast in their explanations, which validates our manipulation. Finally, people’s causal assignments are reflected in the type of explanation given in that contrast and property explanations were associated with biased causal assignments, whereas relational explanations were associated with unbiased assignments. In the discussion, we pick up the normative questions of whether or not these contrast effects constitute a bias in causal reasoning.

## INTRODUCTION

“Is the fact that a piece of wood floats on water basically due to(a) the piece of wood or (b) the water?”

Confronted with this question, about 70% of the German participants in two studies respond “it is due to the wood” (Beller et al., 2009; Bender and Beller, 2011). When asked to explain their choice, the majority of these participants mentioned physical concepts like weight or density, which they know from their physics education, but they ascribed them exclusively to one of the two entities (Beller et al., 2009). Only a few participants noted that, of course, the two entities are equally involved in bringing about the effect of floating. It’s the *relation* of the densities of both, wood and water, that counts. In assigning a stronger causal weight to the piece of wood, people thus ignore the relational nature of the event and the inherent symmetry that the wood contributes to the floating to exactly the same extent as the water.

This kind of weighting in symmetric physical settings is not unusual; we find it for different physical events and in different cultures (e.g., ; Peng and Knowles, 2003; Beller et al., 2009; Bender and Beller, 2011)^1^. Across these studies, the direction and the strength of the effect vary considerably, but identifying the sources for this variability is just in its beginning. So far, the following factors were suggested: the presentation format of the task, that is whether an event is presented visually (Peng and Knowles, 2003; White, 2007) or verbally (Bender and Beller, 2011); the response format, that is whether participants are asked for responsibility assignments (Morris and Peng, 1994; Bender and Beller, 2011) or for explanations (Peng and Knowles, 2003); particular linguistic features such as agent and patient roles (Beller et al., 2009; Mayrhofer and Waldmann, 2014); and framing processes that define what to consider as the figure and what as the background of a scene (Bender and Beller, 2011).

This plurality of factors resonates quite well with what we know from research on causal judgments in general, namely that such judgments can be influenced in various ways: by the familiarity, imageability, and believability of cause and effect relationships (Fugelsang and Thompson, 2000, 2001; Fugelsang et al., 2006), by linguistic cues and the specific causal content (Kuhnmünch and Beller, 2005), by the broader communicative context (Hilton, 1990), by goal-relevant information (Hilton and Knibbs, 1988), by knowledge about possible causal mechanisms (Koslowski et al., 1989; Ahn et al., 1995; Fugelsang and Thompson, 2003; Catena et al., 2008), or by the perceived spatial and temporal aspects of the physical situation (Einhorn and Hogarth, 1986; and see also Buehner and Humphreys, 2010).

In this paper we focus specifically on one modulating factor: *contrast situations* that, in our examples, are formally irrelevant for the solution, but foreground one of the factors in question and thus frame the task in different ways.

### CAUSAL JUDGMENTS AND CONTRAST SITUATIONS

When thinking about whether the piece of wood or the water is responsible for the floating, people establish a “causal field” (Mackie, 1986), which includes all factors they regard as relevant, and then they pick up and foreground one (or more) factors from this field as “causes” for the specific event.

Different kinds of processes contribute to this foregrounding: in some cases, one entity catches people’s attention due to particularly salient features (e.g., Duval, 1972) or because it results from human actions and thus appears controllable (White, 1995; Sloman and Lagnado, 2005). In other cases, the temporal sequence of events is regarded as crucial (e.g., Mill, 1961), or assumptions about what is abnormal as compared to a normal course of events (Hart and Honoré, 1959; Hilton and Slugoski, 1986; Kahneman and Miller, 1986), or what is relevant for an addressee as compared to his or her state of knowledge (Hilton, 1990; Hilton and Erb, 1996). In line with attribution theory and the notion of *man as a scientist* (Heider, 1958; Kelley, 1973), comparing situations in which one factor is present versus absent or modified while other factors are held constant provides another important strategy for selecting what to consider as “the cause,” as suggested by covariation-based models of causal reasoning (e.g., Cheng and Novick, 1990, 1992; Cheng, 1997; White, 2002; Buehner, 2005; Perales and Shanks, 2007).

Some of these foregrounding processes build on an explicit reference to a contrasting situation (cf. Hesslow, 1983). This mechanism can also be applied to our wood-and-water example: here, people might easily think of entities other than wood that either float or sink in water. Comparing the wood to these entities foregrounds the wood, while the water represents the background. As a consequence, people would favor the wood over the water as the causative element, and in explaining the floating they would think about which properties characterize wood in contrast to non-floating objects rather than about the relation between the critical elements wood and water.

Our survey on causal judgments in symmetric physical settings (Bender and Beller, 2011) provided some support for this argument. In one scenario, participants were asked in a “no contrast” baseline condition to decide whether “the fact that CO_2_ stays down in air is due to the CO_2_ or the air.” We then added a contrast situation that foregrounds either the *floater* (“*Helium* rises in air. Is the fact that CO_2_ stays down in air due to …?”) or the *medium* (“CO_2_ rises in *water*. Is the fact that CO_2_ stays down in *air* due to …?”). From a physical point of view, these contrast situations are not relevant for answering the question; what counts is the relation between the two critical entities CO_2_ and air. Nevertheless, the contrasts foreground one of the factors and may thus lead people to think about differences between two situations: why does *CO*_*2*_ stay down in air, whereas *helium* rises? Or: why does CO_2_ stay down in *air*, whereas it rises in *water*? By pointing explicitly to one of the factors as a “difference maker,” the contrast situation should directly affect people’s responses: they should prefer the CO_2_ as causative in the first case, and the medium air in the second case.

**FIGURE 1:**
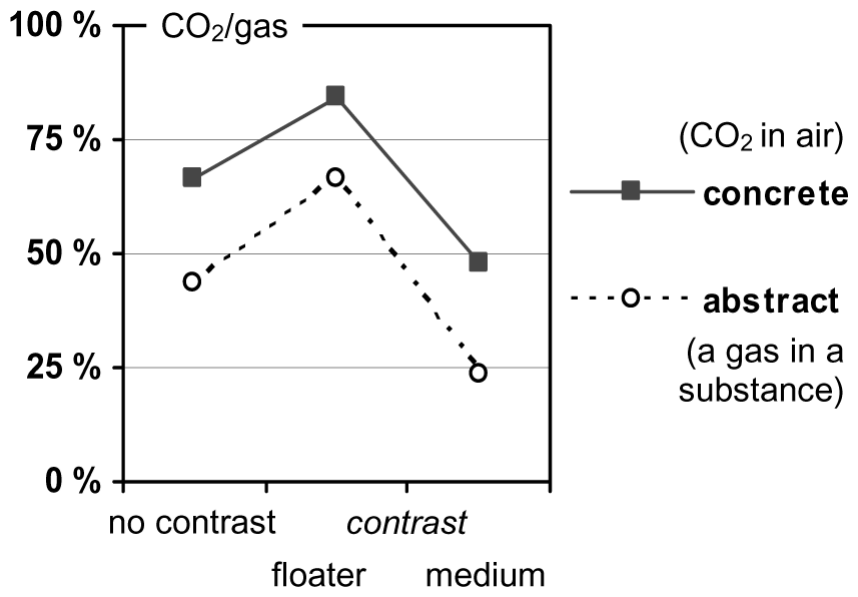
**Preference for “CO_**2**_”/“gas” as the prime cause (from Bender and Beller, 2011; settings (E) and (F); German sample, *N*= 93)**.

The findings from Bender and Beller (2011) support this argument as illustrated in **Figure 1**. There, the results for the three “CO_2_ in air” tasks are represented as *concrete* tasks and compared to analogous *abstract* versions thereof (which will be explained later on). In the no-contrast condition, a moderate imbalance was observed: 66.7% of the participants indicated the gas CO_2_ as causative. The preference for the gas CO_2_ increased if the floater was contrasted (84.0% CO_2_), and it decreased if the medium was contrasted (47.8% CO_2_).

This finding, however, is restricted in three ways. The first concerns the breadth of the results. From the nine physical settings included in our 2011 survey, only one was devoted to this kind of contrast effect. The second restriction is more severe as it concerns how we assessed people’s causal assignments. With one exception, all of our tasks in that survey used the forced-choice format as illustrated in the examples above. The main reason for doing so was that we wanted to prevent participants “from simply activating their formal knowledge acquired in school” (Bender and Beller, 2011, p. 9) and thereby to “tap into deeper, folk-theoretical convictions” (p. 4). While we are still convinced that the forced-choice responses reflect people’s spontaneous assignments of causality and are thus sufficient to detect such weighting effects, it is true that this format leaves participants with no choice other than to give a biased judgment. By excluding the possibility to indicate that the two entities in question contribute equally to the effect, the forced-choice responses might not do justice to people’s physical convictions and might inflate the imbalance. Finally, we so far collected assignment data only and thus focused on *which* decision people take, but not on *why* they take a specific decision, that is, how they explain their causal assignments.

### GOALS OF THE STUDY

In the following, we present two experiments that aim at generalizing and validating the contrast effect from our previous survey (Bender and Beller, 2011). The physical settings and the assessment methods were chosen so as to allow for a direct comparison with previous data. The first experiment maintains the response format (forced-choice), but uses a different physical relation: “wood floating on water.” The result can inform us about whether or not the effect generalizes to another physical setting. By contrast, the second experiment maintains the original physical relation (“CO_2_ staying down in air”), but combines it with a different response format (an analog rating scale). This time, the result can inform us about the extent to which the response format contributes to the effect.

We hypothesize that the contrast effect generalizes to different physical settings. If participants take the contrasting situations into account, this should affect their causal assignments as compared to a baseline task without such a contrast, and it should be reflected in how they explain the physical event in question. To get a spontaneous evaluation of which factor is considered to be the main cause, we first ask for a causal assignment, and then for an explanation of the decision. The response format might have a differentiating effect: participants’ causal assignments might overall be less biased with the rating format than with the forced-choice format as only the former allows them to consider the relational character of the task appropriately. This might also be reflected in people’s explanations.

Finally, in order to be able to assess the extent to which possible content-specific associations with the involved entities (wood/water and CO_2_/air, respectively) contribute to participants’ assignments and give rise to *content effects* beyond the type of physical interaction (as reported, for example, by Beller and Kuhnmünch, 2007, and Beller et al., 2009), we implemented the physical relations (e.g., “floating”) in two versions each: in a *concrete* version (e.g., instantiated by “wood” and “water”) and an *abstract* version (e.g., instantiated by “an object” and “a liquid”).

## EXPERIMENT 1

This experiment aimed at generalizing the contrast effect from Bender and Beller (2011) to a different physical setting, while the response format (forced-choice) was the same as in our original survey. For the baseline condition with an explicit contrast situation, we expected a preference for the floating object as observed in a previous study (Beller et al., 2009).

### METHOD

#### Participants

A total of 111 students (30 male, 81 female) from the University of Freiburg (Germany) volunteered to participate in the experiment. The mean age was *M* = 22.7 years (SD = 4.61; range: 18–45 years).

#### Materials

Six causal assignment tasks were constructed by crossing three conditions with different contrast situations (no contrast, contrasting object vs. contrasting liquid) with two content scenarios (concrete vs. abstract). All tasks referred to the same physical relation (an object floating on a liquid) and called for a forced-choice decision on which of the two entities involved is responsible for the floating.

The three concrete tasks read as follows:

*No contrast:* The fact that a piece of wood floats on water is basically due to ...

□ the piece of wood.
□ the water.

*Contrasting object:* A piece of coal sinks in water. The fact that a piece of wood floats on water is basically due to ...

□ the piece of wood.
□ the water.

*Contrasting liquid:* A piece of wood sinks in oil. The fact that the same piece of wood floats on water is basically due to ...

□ the piece of wood.
□ the water.

The corresponding abstract tasks read as follows:

*No contrast:* The fact that an object floats on a liquid is basically due to ...

□ the object.
□ the liquid.

*Contrasting object:* An object A sinks in a liquid. The fact that a different object B floats on the same liquid is basically due to ...

□ object B.
□ the liquid.

*Contrasting liquid:* An object sinks in a liquid A. The fact that the same object floats on a different liquid B is basically due to ...

□ the object.
□ liquid B.

Participants were instructed to make their assignment spontaneously and to mark one option. Each task was followed on a separate page by the request to briefly explain their decision (“Bitte begründen Sie kurz Ihre Entscheidung.”). All materials were presented in German and were pretested for comprehensibility.

#### Procedure and design

The experiment was part of a larger paper-and-pencil questionnaire study with different kinds of reasoning tasks (none of the other tasks, however, was on a causal topic). The part of the questionnaire relevant here consisted of four pages: (i) first causal assignment task, (ii) first explanation, (iii) second assignment task, and (iv) second explanation.

For each questionnaire booklet, one of the three concrete contrast conditions was paired with one of the three abstract contrast conditions (in first or second position, respectively). From the 18 possible combinations of conditions, we implemented all possibilities except those with a no-contrast condition in the second position. The reason for this restriction was that we wanted to prevent a possible carry-over of contrast information from the more complex conditions with a contrasting situation to the simpler no-contrast conditions. We thus had six pairings with a concrete task in first position and six pairings with an abstract task in first position. The order of the answer options (object first vs. liquid first) was balanced within the pairings (across the two tasks) and also across the pairings. We prepared an appropriate number of the different booklet versions so that, in the end, the different conditions of both the concrete and the abstract scenario occurred with (almost) equal frequencies across participants. Finally, participants were assigned randomly to the prepared questionnaires.

### RESULTS AND DISCUSSION

**FIGURE 2:**
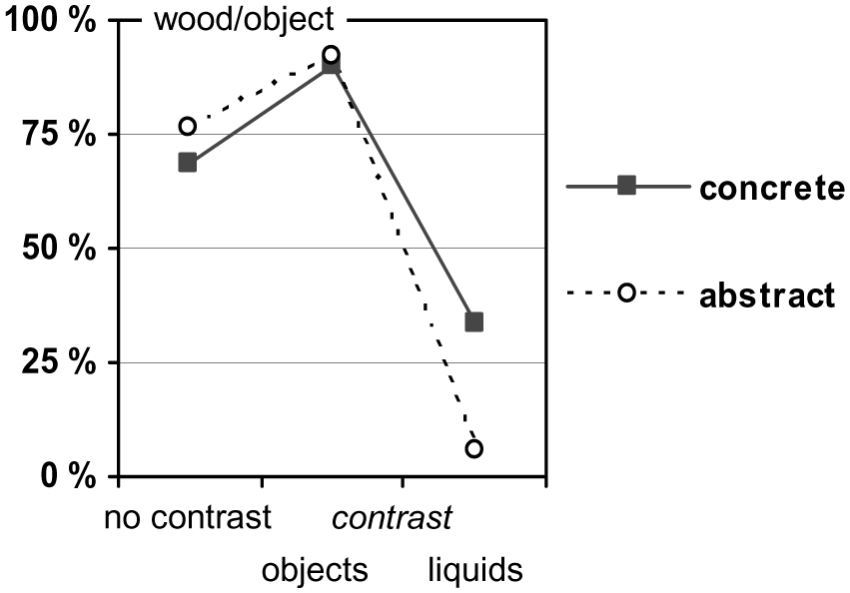
**Preference for “a piece of wood”/“the object” as the prime cause in Experiment 1**.

Participants’ causal assignments are presented in **Figure 2** with values above 50% indicating a more frequent choice of the floater as causative (i.e., the piece of wood or the object, respectively), and values below 50% indicating a more frequent choice of the carrier (i.e., the water or the liquid). The data pattern confirms both the predicted preference for the floater in the no-contrast baseline and the effects of the contrasting situations.

A four-way log-linear analysis was conducted (Kennedy, 1992) with the causal assignments (floater vs. carrier) as dependent variable, “content,” (abstract vs. concrete) and “contrasting situation” (no contrast vs. contrasting objects vs. contrasting liquids^2^) as independent variables, and “position of task” (first vs. second position), and “order of answer options” (object first vs. liquid first) as control variables. The analysis revealed that the two control variables were not necessary to explain the data; they could be removed from the model without losing the fit (fit of the resulting model “content × contrasting situation”: *G^2^* = 22.1, *df* = 14, *p* = 0.077)^3^. As expected, the causal assignments varied strongly across the three contrasting conditions (main effect “contrasting situation”: *G^2^* = 90.4, *df* = 2, *p* < 0.001). No main effect “content” occurred (*G^2^* = 0.966, *df* = 1, *p* = 0.326), but an interaction “content × contrasting situation” (*G^2^* = 8.7, *df* = 2, *p* = 0.013), which was mainly triggered by the contrasting liquids condition, with the contrast effect being more pronounced in the abstract version than in the concrete version.

The concrete tasks revealed the following results: in the no-contrast condition, participants preferred the wood as causative for the floating (68.4% wood vs. 31.6% water; χ^2^ = 5.2, *df* = 1, *n* = 38, *p* = 0.023). The preference for wood increased if the object was contrasted (90.0% wood vs. 10% water; *n* = 40), and it changed to the water if the liquid was contrasted (33.3% wood vs. 66.7% water; *n* = 33). The causal assignments in the two contrasting conditions differed from one another (χ^2^ = 25.3, *df* = 1, *n* = 73, *p* < 0.001), and also from the no-contrast condition (χ^2^ = 5.6, *df* = 1, *n* = 78, *p* = 0.018, and χ^2^ = 8.7, *df* = 1, *n* = 71, *p* = 0.003, respectively).

An analogous pattern was found for the abstract tasks: in the no-contrast condition, participants again preferred the object as causative for the floating (76.3% object vs. 23.7% liquid; χ^2^ = 10.5, *df* = 1, *n* = 38, *p* = 0.001). The preference for the object increased if the object was contrasted (92.1% object vs. 7.9% liquid; *n* = 38), and it changed to the carrying liquid if the liquid was contrasted (5.7% object vs. 94.3% liquids; *n* = 35). The causal assignments in the two contrasting conditions differed from one another (χ^2^ = 54.4, *df* = 1, *n* = 73, *p* < 0.001). Compared to the no-contrast condition, the increase triggered by the object contrast was only marginally significant (χ^2^ = 3.6, *df* = 1, *n* = 76, *p* = 0.059), whereas the decrease triggered by the liquid contrast was significant (χ^2^ = 37.2, *df* = 1, *n* = 73, *p* < 0.001).

These findings replicate the biased causal assignments for the relation “wood floats on water” in the absence of explicit contrasts (as described by Beller et al., 2009, and Bender and Beller, 2011), it demonstrates the contrast effect for this setting, and it suggests that the two effects are only weakly affected by the specific types of entities involved.

**FIGURE 3:**
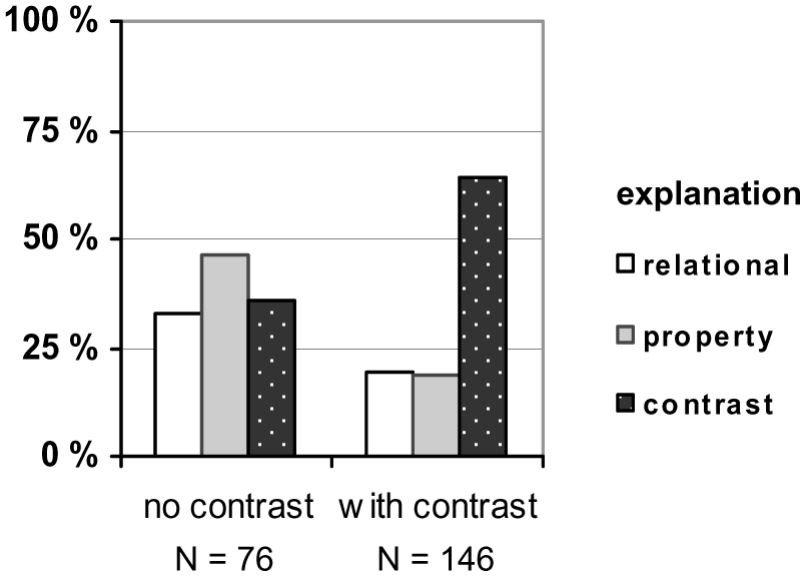
**Explanations of the causal assignments in Experiment 1 (in %; *N* represents the sum of concrete and abstract tasks)**.

In order to shed light on participants’ rationale for their decisions, we had asked them for explanations. Their statements were classified into four categories: “relational,” “property,” “contrast,” and “others.” A statement was classified as *relational* if it mentioned the two relevant factors as being equally causative (e.g., “Actually, both are responsible”) or if it explicitly referred to an interaction between these two factors (e.g., “The density of wood is lower than the density of water”). Some explanations focused on an individual *property* either of the floating object (e.g., “The wood contains air” or “density of the wood”) or of the carrier liquid (e.g., “due to the buoyancy of the water”). Finally, a statement was classified as referring to a *contrast* if it compared the floating object to another, non-floating object (e.g., “Coal is heavier than wood”), if it compared the carrier liquid to another, non-carrying liquid (e.g., “Oil is lighter than water”), or if it referred to a comparison of situations in abstract terms (e.g., “The condition that was changed, the liquids, is the deciding factor”). The frequencies of the different types of explanations were aggregated for the two contrast conditions and across concrete and abstract tasks, as the differences between the two content versions were only small. The results are presented in **Figure 3**.

The explanation data revealed two main findings. First, in the no-contrast tasks, participants gave all three types of explanations with similar frequencies. Explanations in terms of individual properties of floater and liquid slightly prevailed, mostly referring to density and weight. This also means that some participants used the contrast strategy to explain the floating without being triggered by an explicit contrast situation. Second, compared to this baseline, relational explanations decreased from 32.9 to 19.2% when a contrasting situation was introduced (χ^2^ = 5.2, *df* = 1, *N* = 222, *p* = 0.023), and so did property explanations; they decreased from 46.1 to 18.5% (χ^2^ = 18.7, *df* = 1, *N* = 222, *p* < 0.001). On the other hand, contrast explanations increased from 35.5 to 64.4% (χ^2^ = 16.8, *df* = 1, *N* = 222, *p* < 0.001). As a manipulation check, this finding validates our experimental setting, and it supports our argument that a contrast situation leads participants to focus on the entities involved in this contrast.

All in all, Experiment 1 was successful in extending the contrast effect on a new symmetric physical setting. However, as in our previous studies, we used a response format that forced participants to take a dichotomous decision. With this format, we intended to outmaneuvre possible effects of school education and to tap into deeper, folk-theoretical convictions, but it could be argued that this format also has skewed participants’ responses in an unintended way and beyond their actual preferences. This concern is addressed in the next experiment.

## EXPERIMENT 2

In order to assess the influence of the response format, Experiment 2 combines the original physical setting from Bender and Beller (2011)—CO_2_ staying down in air—with an analog rating scale that allows participants to allocate the *relative* causal effectiveness of the two entities in question. While the possibility to indicate the physically adequate relational answer might generally reduce the imbalance in participants’ causal assignments, we nevertheless expected to find an effect of contrast situations.

### METHOD

#### Participants

A total of 128 students (32 male, 96 female) from the University of Freiburg (Germany) volunteered to participate in the experiment (there was no overlap with participants from Experiment 1). The mean age was *M* = 22.5 years (SD = 4.71; range*:* 18–45 years).

#### Materials

Six causal assignment tasks were constructed by crossing three conditions with different contrast situations (no contrast, contrasting floater vs. contrasting medium) with two content conditions (concrete vs. abstract). All tasks referred to the same physical relation (a gas staying down in a substance) and allowed participants to indicate the relative causal effectiveness of the entities involved by means of an analog rating scale of 10 cm length.

The three concrete tasks read as follows:

*No contrast:* The fact that CO_2_ stays down in air is basically due to …
the CO_2_ |———————————————| the air.
*Contrasting floater:* Helium rises in air. The fact that CO_2_ stays down in air is basically due to …
the CO_2_ |———————————————| the air.
*Contrasting medium:* CO_2_ rises in water. The fact that CO_2_ stays down in air is basically due to …
the CO_2_ |———————————————| the air.

The corresponding abstract tasks read as follows:

*No contrast:* The fact that gas G stays down in substance S is basically due to …
gas G |———————————————| substance S.
*Contrasting floater:* Gas X rises in substance S. The fact that gas G stays down in substance S is basically due to …
gas G |———————————————| substance S.
*Contrasting medium:* Gas G rises in substance X. The fact that gas G stays down in substance S is basically due to …
gas G |———————————————| substance S.

Participants were instructed to make their assignment spontaneously. Each task was followed on a separate page by the request to briefly explain their decision from the previous task (“Bitte begründen Sie kurz Ihre Entscheidung aus der vorigen Aufgabe.”). All materials were presented in German and were pretested for comprehensibility.

#### Procedure and design

These were analogous to Experiment 1. However, instead of the order of the response options, the polarity of the rating scale was balanced (CO_2_/gas G marking the left side vs. the right side of the scale).

### RESULTS AND DISCUSSION

**FIGURE 4:**
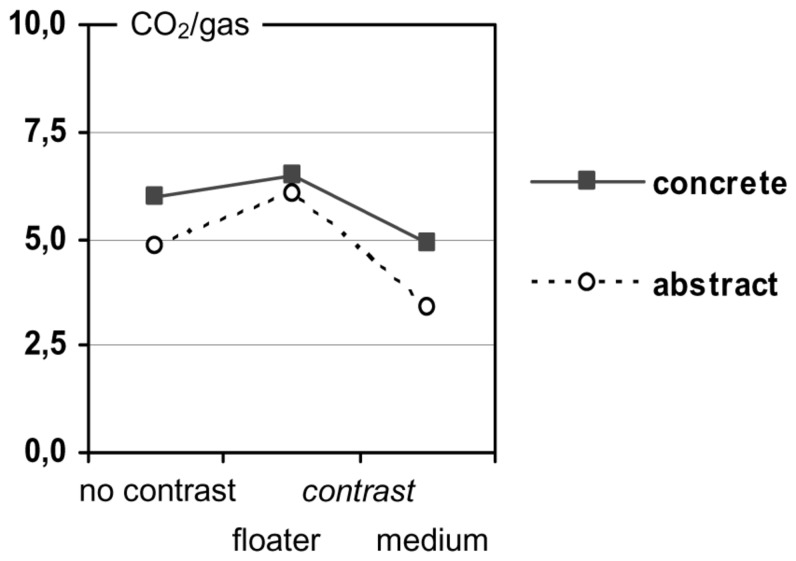
**Preference for “CO_**2**_”/“the gas” as the prime cause in Experiment 2**.

Participants’ causal assignments were coded by measuring their marks on the rating scale (accurate to 0.5 mm) ranging from 0 (the mark was right on the endpoint labeled with “air/substance”) to 10 (the mark was right on the endpoint labeled with “CO_2_/gas G”). Therefore, values above 5.0 indicate a stronger causal role of the floater (CO_2_/gas G), and values below 5.0 indicate a stronger causal role of the medium (air/substance S). The results are presented in **Figure 4**. The data patterns of the concrete and the abstract tasks generally replicate the findings from Experiment 1 (**Figure 2**) and from our previous survey (**Figure 1**), albeit in a less extreme manner.

In the first step, we checked the rating data for possible position effects. To this end, two analyzes of variance were conducted (one for the concrete tasks and one for the abstract tasks) with “position of task” (first vs. second position) and “contrasting situation” (contrasting floater vs. medium) as independent variables. We did not include the no-contrast conditions in these analyzes as the respective tasks were presented always in first position. As the two analyzes did not indicate any position effects (for all effects with “position”: *F*s(1,80) < 3.33, *p*s > 0.072), it seemed safe to run the subsequent analyzes without this factor.

In the second step, we analyzed the ratings with “contrasting situation” (now all three types) and “polarity of the rating scale” as independent variables, again separately for concrete and abstract tasks. The two analyzes did not indicate any polarity effects [for all effects with “polarity”: *F*s(1,122) < 1.75, *F*s(2, 122) < 0.35, *p*s > 0.189], but revealed main effects of the contrasting situation [concrete content: *F*(2,122) = 3.72, *p* = 0.027; abstract content: *F*(2,122) = 13.44, *p* < 0.001].

The concrete tasks exhibited the following results: in the no-contrast condition, we found an imbalanced causal assignment: participants preferred the gas (CO_2_) over the medium (air), with *M* = 5.94 (*n* = 44) being significantly different from the balanced value 5.0; *t*(43) = 2.33, *p* = 0.025. The causal assignment changed as expected, when a contrasting situation was added. Participants regarded CO_2_ as more causative if the gas was contrasted (*M* = 6.45, *n* = 42), but as less causative if the medium was contrasted (*M* = 4.92, *n* = 42). The average ratings in the two contrasting conditions differed from one another (LSD = 1.53, *p* = 0.008), but not from the no-contrast baseline condition (LSD = 0.51, *p* = 0.372, and LSD = 1.02, *p* = 0.073, respectively).

A slightly different pattern was found for the abstract tasks: this time, the no-contrast condition indicated a quite symmetric causal assignment with *M* = 4.83 (*n* = 44), not being significantly different from the balanced value 5.0; *t*(43) = -0.523, *p* = 0.604. But, again, the assignments changed as expected, when a contrasting situation was added: participants regarded the gas as more causative than the medium if the gas was contrasted (*M* = 6.07, *n* = 42), but as less causative if the medium was contrasted (*M* = 3.38, *n* = 42). The average ratings in the two contrasting conditions differed from one another (LSD = 2.69, *p* < 0.001), and also from the no-contrast condition (LSD = 1.24, *p* = 0.017, and LSD = 1.45, *p* = 0.006, respectively). Compared to the concrete tasks, responses in the abstract tasks showed generally lower values, indicating a stronger weight on the medium as causative [abstract*: M* = 4.76; concrete*: M* = 5.77; *t*(127) = -2.876, *p* = 0.005, paired samples test], and the influence of contrasting situations seemed to be stronger.

**FIGURE 5:**
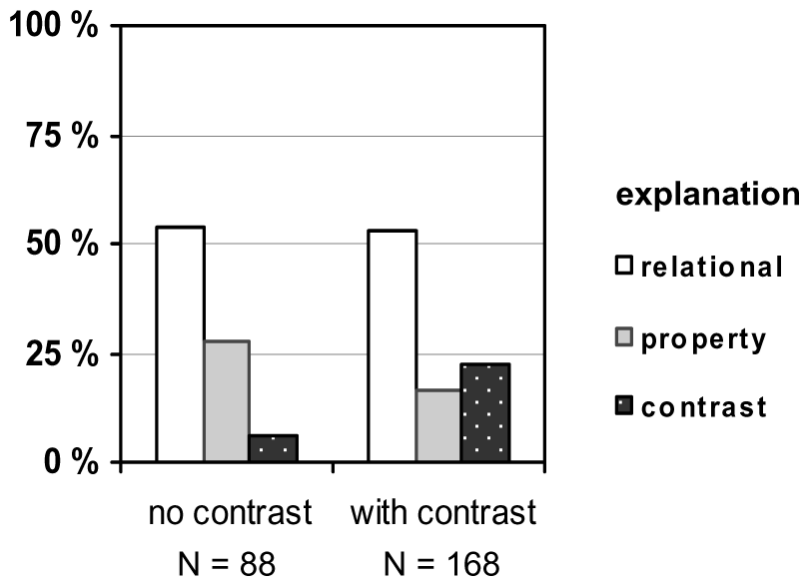
**Explanations of the causal assignments in Experiment 2 (in %; *N* represents the sum of concrete and abstract tasks)**.

Next, we categorized people’s explanations of their causal assignments as described for Experiment 1. The results are shown in **Figure 5**, aggregated across concrete and abstract tasks as the differences between the two content versions were again only small. The proportion of relational explanations was clearly higher than in Experiment 1 and did not differ between the no-contrast and the contrasting conditions (53.4 and 53.0%, respectively; χ^2^ = 0.004, df = 1, *N* = 256, *p* = 0.947). But, similar to Experiment 1, property explanations decreased from 27.3 to 16.1% (χ^2^ = 4.5, *df* = 1, *N* = 256, *p* = 0.033) and contrast explanations increased from 5.7 to 22.0% (χ^2^ = 11.2, df = 1, *N* = 256, *p* = 0.001) when a contrasting situation was available.

The increase of relational explanations, and correspondingly the decrease of the other types of explanations, is most probably a direct consequence of the differences in the response format (as is the overall less extreme causal assignments compared to Experiment 1): with the forced-choice format, participants have to commit themselves to one entity as causative in the assignment task, and later on have to explain this decision regardless of whether or not they are able to figure out the physically appropriate response. With the rating-format, however, participants can indicate in the assignment task that both entities are equally causative, and are therefore not restricted later on to explain an either-or decision, but might draw on their physical knowledge instead.

**FIGURE 6:**
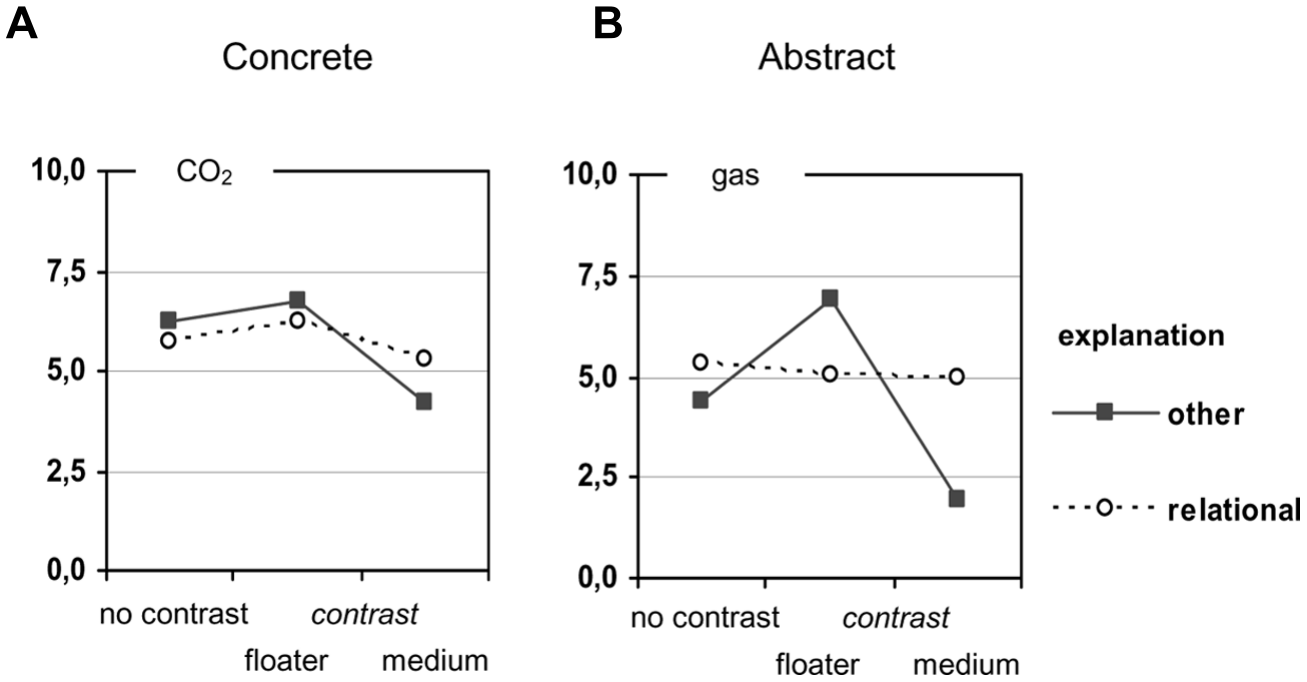
**Preferences in Experiment 2 **(A)** for “CO_**2**_” as the prime cause in the concrete scenarios and **(B)** for “the gas” in the abstract scenarios, depending on the type of explanation**.

If we assume that those participants providing an adequate relational explanation did not base their (preceding) causal assignment on a contrasting situation, while the other participants did include a contrast in their reasoning in one or the other way, then we would expect the contrast effect to disappear for the first group and to be more pronounced for the second. To test this prediction, we re-analyzed the ratings by means of two analyzes of variance (one for the concrete tasks and one for the abstract tasks) in which we included the “type of explanation” (relational vs. all other) as an independent variable in addition to the “contrasting situation” and the “polarity of the rating scale.” As before, these analyzes revealed no polarity effects, but main effects of the contrasting situation [concrete content: *F*(2,116) = 4.51, *p* = 0.013; abstract content: *F*(2,116) = 13.91, *p* < 0.001]. With regard to the type of explanation, we got different results depending on the task content as indicated in **Figure 6**.

For the concrete tasks, we did not find any effect of the type of explanation [largest *F*(1,116) = 3.11, *p* = 0.080]. The contrast effect appears to be weaker for participants with relational explanations than for those with non-relational explanations, but the interaction was not significant [*F*(2,116) = 1.47, *p* = 0.235]. If we take a more qualitative perspective and count how many participants placed their rating in the middle of the scale, defined as the interval [4.5; 5.5], we find nonetheless a striking difference: among those 75 participants with relational explanations, 64% placed their rating in the middle of the scale, but only 9% among the 53 participants with non-relational explanations.

For the abstract tasks, the interaction “type of explanation × contrasting situation” was significant [*F*(2,116) = 13.48, *p* < 0.001]. Here, the causal assignments of the participants with relational explanations were nearly perfectly balanced in each of the three contrast conditions, indicating that the overall contrast effect reported in **Figure 4** was mainly driven by the participants with non-relational explanations. This is also reflected by the qualitative inspection: among those 61 participants with relational explanations, 77% placed their rating in the middle of the scale, but only 22% among the 67 participants with non-relational explanations. These findings are in line with our hypothesis that the unbiased causal assignments result from not referring to a contrast situation, as suggested by participants (*post hoc*) explanations.

On the whole, Experiment 2 was successful in validating the contrast effect with a less restrictive response format, and the inspection of people’s explanations provided further evidence for the assumed contrasting mechanisms. Participants’ causal assignments appeared to be fairly well reflected in their later explanations, but the correspondence was not perfect. In about 30% of the cases, in which participants apparently *knew* that both entities are equally involved in bringing about the effect, they still had given one of the entities a stronger weight.

## GENERAL DISCUSSION

Physical settings in which two entities interact to bring about an effect are symmetric, and, from that point of view, responsibility should be assigned in a balanced manner. Often, however, people make imbalanced causal assignments by giving one of the factors a stronger weight. Against this background, the goals of this paper were twofold: we wanted to validate the method of assessing such weighting effects, and to put the contrast effect from Bender and Beller (2011) on a broader empirical basis. Starting from the one relevant example from our 2011 survey, Experiment 1 combined a different physical relation with the original response format (forced-choice) thereby broadening the kinds of physical settings considered, whereas Experiment 2 combined the original physical relation with a new response format (an analog rating scale) thereby providing us with insights into the role of the response format.

Taken together, the experiments yielded four main results: first, in three of the four conditions without an overt contrast we observed biased causal assignments. These were more pronounced in Experiment 1 with the forced-choice format than in Experiment 2 with the rating format, and they replicate previous findings quite well (Beller et al., 2009; Bender and Beller, 2011). The decision on which entity to regard as causative is not without consequences, as we then tend to overestimate the strength and importance of the cause for bringing about the effect (White, 2006, 2007). White (2006) considers such cause-effect asymmetries as a general feature of human causal reasoning that affects most of what people perceive and believe regarding the causal underpinning of the world.

Second, the response format clearly made a difference. Giving participants the possibility to indicate the relative causal effectiveness of the two entities in question, instead of forcing them into a decision, resulted generally in more balanced causal assignments and more relational explanations. This does not imply, however, that all biases automatically disappear with this kind of assessment. The concrete no-contrast task in Experiment 2 is a case in point, showing a small but nevertheless stable imbalance (see **Figure 4**). The less extreme responses with the rating scale may have two reasons. On the one hand, the rating format allows people to directly express that the two entities are equally important, and many of the participants in Experiment 2 did so. In fact, 45% of all ratings fell into the interval [4.5; 5.5] that represents the middle of the scale. On the other hand, even those participants who gave one entity a stronger weight often hesitated to discount the other entity completely; only 10.5% of all participants marked an endpoint of the scale.

Third, irrespective of the response format, the two experiments confirm that people are sensitive to contrast information. Experiment 2 replicates the general data pattern from Bender and Beller (2011), while Experiment 1 establishes the contrast effect for a different physical setting and thus broadens the empirical basis of this phenomenon. In both cases, contrast effects were found both for the concrete and the abstract task versions. The contrast situation draws people’s attention to the contrasting factor and lets them weight this factor more strongly. Even in our baseline conditions without an explicit contrast, some of the participants explained their causal assignment by providing a contrast. Our findings thus underline the importance of contrast situations in causal reasoning and judgment.

Finally, the results also provide some indication for content effects (Beller and Spada, 2003; Beller et al., 2009; Bender and Beller, 2011). On the one hand, participants’ causal assignments varied with the specific entities involved in a physical relation, at least to some extent. For instance, when reasoning about why a gas is staying down in a substance, participants generally gave the gas a stronger weight in the concrete condition than in the abstract condition, irrespective of the response format (see **Figures 1 and 4**). This difference suggests that specific associations with the one or the other entity involved in the interaction—for example, that CO_2_ is a particularly dense gas—can pave the way for a causal weighting in a specific direction. On the other hand, the type of interaction plays an additional role, as an inspection of **Figures 1 and 2** suggests. Here, with the response format held constant, the strength of the weighting effects clearly varies with the type of physical relation.

Two possible limitations need to be addressed though: first, we had instructed our participants to make their causal assignments *spontaneously*. This should have led them into a more intuitive, “system 1” based mode of reasoning, instead of a more deliberate, “system 2” based mode (Evans, 2003, 2009), and this was intended for the very same reason that we used the forced-choice response format: we actually wanted to tap into people’s folk-theoretical convictions instead of simply activating some physical knowledge acquired in school. While such a suppression of deliberate thinking might contribute to inflate the number of imbalanced causal assignments, it does not invalidate the consequences of basing causal judgments on contrasting situations as attested by the analysis of the explanations. Moreover, we have no clear indication to which extent participants’ judgments were in fact spontaneous, as they were granted as much time as they needed to fill in the questionnaire. The effect of this instruction should thus not be overrated. To properly assess this, however, the present experiments could be extended in future research so as to compare our spontaneity instruction that triggers system-1-based thinking with an instruction that demands thinking through the problem carefully and thus triggers system-2-based thinking. Second, as might have been noticed, the concrete and the abstract version of the gas setting in Experiment 2 were not exactly parallel. The concrete version states that “CO_2_ stays down in air” (with air being a mixture of gases), whereas the abstract version states that “gas G stays down in substance S” and thus introduces a different kind of medium (the substance) that might or might not be aeriform. The reason for using the term “substance” was that we wanted to make clear that one contrast really involves two kinds of medium (substance X and S). Being uncertain about what is meant by “a substance” might reduce biased causal assignments, particularly in the no-contrast baseline condition. Once an explicit contrast situation is introduced, a rational for the decision is introduced so that the uncertainty no longer matters.

Finally, two broader questions remain to be answered, a normative one and a psychological one: should we consider the contrast effect in symmetric physical settings a *bias*? And, what is the reason for the imbalanced causal assignments in the conditions without an explicit contrast?

The first question concerns the tension between what (many) people actually *do* in the contrasting conditions and what they *should* do from a physical point of view. The normative answer is clear: as the physical relations in question are symmetrical, the two entities involved should be weighted equally strongly—*irrespective* of the included contrast situation. To answer the question why a piece of wood is floating on water, it is simply not relevant whether a piece of coal does or does not float on water. Framed in this way, it may appear as a bias if people take the contrast into account while not recognizing the symmetry of the physical relation. And, as documented in people’s explanations, the contrast situations distract at least some of them from thinking about the properties of the two relevant entities. But there is good reason for considering such contrasts. Not only does the contrast strategy comply with Grice’s principle of relevance in conversation (Grice, 1975, 1989) in that all information given in a task should be considered important for its solution. This may have generated a kind of demand characteristics to include the contrast in the reasoning process. Comparing situations is also a typical strategy in causal reasoning, which can provide important hints for the search of a causal explanation of the phenomenon in question. In other words: thinking about the difference between a piece of wood and a piece of coal in their relation to water is indeed an important step toward understanding the phenomenon of floating.

The second question on how the imbalanced causal assignments in the no-contrast conditions can be explained is more difficult to answer. Here, participants’ explanations—albeit as *post hoc* rationalizations not fully conclusive—might again provide us with some insights. A substantial number of participants gave a correct relational explanation such as “The density of CO_2_ is higher than the density of air,” but failed to recognize the symmetry of this relation and ascribed the relevant property only to one entity (mostly to CO_2_ in this specific example). Other participants seemed to focus from the outset on specific properties that came to their mind for one of the entities (as one type of content effect), for example, “wood contains air,” “the surface tension of water,” or “buoyancy exerted by water.” We also found arguments that build on contrasting situations, for example, comparing abnormal to normal states (e.g., “normally, gases rise in a substance”) or on comparisons of observed to hypothetical states (“If the gas was lighter, then it would rise”). These are indicative for the relevance of norms (e.g., Hart and Honoré, 1959; Hilton and Slugoski, 1986; Kahneman and Miller, 1986) and for counterfactual reasoning processes (e.g., Byrne, 2002; Mandel et al., 2005). In general, we can assume the choice of contrast situations to be guided by how easily people might think of alternatives (Byrne, 2005). And finally, a small proportion of the participants argued purely linguistically: “The gas is the subject of the sentence.” Corresponding linguistic effects are reported, for example, by Beller et al. (2009).

Taken together, this diversity of explanations suggests that biases in causal assignments are (likely) triggered not by one single rationale, but can be triggered by a host of different cues. The general mechanism involves processes of activating, selecting, and weighting reasons, but we have only just begun to explore this conglomeration—by singling out and varying the influence of some factors or, in other words, by contrasting situations.

## Conflict of Interest Statement

The authors declare that the research was conducted in the absence of any commercial or financial relationships that could be construed as a potential conflict of interest.

## Footnotes

1 A broader discussion of causal reasoning across domains and cultures is provided in the Research Topic *Diversity and universality in causal cognition* published in Frontiers in Cognitive Science (Beller et al., 2014).

2 As the baseline tasks were only presented in first position, four “zero cells” were defined to accommodate the fact that not all possible combinations of the independent variables were instantiated.

3 A log-linear model fits the data if the test statistic does *not* indicate a significant difference between model and data.
